# Multifunctional Vanadium Nitride-Modified Separator for High-Performance Lithium–Sulfur Batteries

**DOI:** 10.3390/nano14080656

**Published:** 2024-04-10

**Authors:** Sen Liu, Yang Liu, Xu Zhang, Maoqiang Shen, Xuesen Liu, Xinyue Gao, Linrui Hou, Changzhou Yuan

**Affiliations:** School of Materials Science & Engineering, University of Jinan, Jinan 250022, China; liusen@stu.ujn.edu.cn (S.L.); zhangxu@stu.ujn.edu.cn (X.Z.); shenmq@stu.ujn.edu.cn (M.S.); liuxs@stu.ujn.edu.cn (X.L.); gaoxinyue@stu.ujn.edu.cn (X.G.); mse_houlr@ujn.edu.cn (L.H.)

**Keywords:** lithium–sulfur batteries, separator modification, lithium polysulfide, vanadium nitride, bifunctional catalytic, kinetic

## Abstract

Lithium–sulfur batteries (LSBs) are recognized as among the best potential alternative battery systems to lithium-ion batteries and have been widely investigated. However, the shuttle effect has severely restricted the advancement in their practical applications. Here, we prepare vanadium nitride (VN) nanoparticles grown in situ on a nitrogen-doped carbon skeleton (denoted as VN@NC) derived from the MAX phase and use it as separator modification materials for LSBs to suppress the shuttle effect and optimize electrochemical performance. Thanks to the outstanding catalytic performance of VN and the superior electrical conductivity of carbon skeleton derived from MAX, the synergistic effect between the two accelerates the kinetics of both lithium polysulfides (LiPSs) to Li_2_S and the reverse reaction, effectively suppresses the shuttle effect, and increases cathode sulfur availability, significantly enhancing the electrochemical performance of LSBs. LSBs constructed with VN@NC-modified separators achieve outstanding rate performance and cycle stability. With a capacity of 560 mAh g^−1^ at 4 C, it exhibits enhanced structural and chemical stability. At 1 C, the device has an incipient capacity of 1052.4 mAh g^−1^, and the degradation rate averaged only 0.085% over 400cycles. Meanwhile, the LSBs also show larger capacities and good cycling stability at a low electrolyte/sulfur ratio and high surface-loaded sulfur conditions. Thus, a facile and efficient way of preparing modified materials for separators is provided to realize high-performance LSBs.

## 1. Introduction

Under the urgent requirement of “carbon neutrality”, energy storage devices are instrumental in expanding the efficiency of energy use and diversifying application areas, and electrochemical energy storage devices are also gaining attention in technology and markets [1,2,3]. Lithium–sulfur batteries (LSBs) are recognized as among the best potential alternative battery systems to lithium-ion batteries, as they are inexpensive, environmentally friendly, and have great energy density of the whole system [4,5]. However, LSBs still have serious problems such as fast capacity decay, short cycle life, and the energy density of actual devices being much lower than the theoretical value [6,7,8]. The inhibition of the transmembrane diffusion of LiPSs, the solution of the lithium dendrite problem, and the overall enhancement of the properties of LSBs are the keys to achieving the process of practical LSBs, which have generated substantial concern among researchers in recent years [9,10,11,12].

In the presence of cathode problems in LSBs, the majority of researchers have made many efforts and adopted various strategies. For example, the confinement of singlet sulfur in the porous and conductive skeleton material [13,14]. The construction of a physical or chemical protection layer on the cathode side and other strategies to suppress the shuttle effect and raise cathode sulfur availability. The problem of lithium dendrite growth on the anode side has been solved through modification of the electrolyte, the introduction of lithophilic materials at the anode, and the use of solid-state electrolytes [15,16,17]. The solid-state electrolyte can also significantly restrict the dissolution of LiPSs, thereby significantly controlling the shuttle effect, in which the presence of S^2−^ in the sulfide solid-state electrolyte can broaden the lithium-ion transport channel with better lithium-ion conductivity, while the enhancement of the interfacial ion mobility rate effectively improves the utilization of the active substance [18,19,20]. In addition, the binder has a significant impact on the performance of LSBs. Sulfur cathodes generate great volume expansion and contraction during the cycling process. Therefore, the binder must have a very good mechanical modulus to buffer the volume change during the cycling period. Binders such as polyvinylpyrrolidone (PVP) and polyethylene oxide (PEO) have been chosen to replace polyvinylidene fluoride (PVDF) to enhance the interaction with the polysulfide and suppress the shuttle effect [21,22,23]. These tactics significantly enhance the performance of LSBs, but the design of cathode and anode structures and the choice of electrolyte and binder usually lead to an increase in cost and a decrease in energy density, limiting the further development of LSBs. As one of the key constituents in the battery system, the separator is not directly involved in the electrode reaction but has a significant influence on the kinetic processes such as ion transport within the battery system and transmembrane diffusion of soluble LiPSs in LSBs, which is the major cause of fast degradation of cathode capacity and passivation of lithium metal anodes [24,25,26,27]. Recently, it has been found that a straightforward modification to the separator could significantly improve the electrochemical characteristics of LSBs [28,29,30]. The modified separator exhibits unique advantages and great potential in speeding up the kinetics in cathode sulfur redox reactions and suppressing the shuttle effect, safeguarding the Li-metal anode and reducing the interfacial transfer resistance [31,32,33,34]. Researchers can modify the separators in ways for different purposes and the process is simple and cost-effective [10,35]. Porous carbon with a high surface area is considered a promising functional material to maximize the physisorption of polysulphides and suppress the shuttle effect, and the porous structure also mitigates the volume expansion of electrochemical reaction products [10,14]. According to the difference between the radius of polysulfide anion and lithium ion, materials with ion-sieving function characteristics are selected to modify the separator. For instance, graphene and conductive carbon skeletons inhibit the transmembrane diffusion of LiPSs through the role of physical domain limitation [36,37,38,39,40]. In addition to the spatial domain-limiting measure, the shuttling effect can also be preceded by enhancing the chemical anchoring of LiPSs to improve the availability of reactive sulfur [41,42]. Heteroatom doping such as nitrogen, sulfur, phosphorus, and boron in carbon or MXene-based materials can effectively enhance the chemisorption of LiPSs [43,44,45,46,47]. Transition metal sulfides, nitrides, and oxides provide efficient chemical anchoring of LiPSs while also improving the reaction kinetics and showing excellent performance in suppressing the shuttle effect [48,49,50,51,52]. However, owing to the physicochemical characteristics of each material, how to maintain an efficient transport channel for lithium ions while effectively blocking LiPSs is the key issue in the research of separator modification toward LSBs [53,54,55].

Here, we prepared VN nanoparticles grown in situ on a nitrogen-doped carbon skeleton (VN@NC) derived from the MAX phase and used it as separator-modified material to improve the electrochemical properties of LSBs. The VN@NC exhibited outstanding electrical conductivity and catalytic properties, provided abundant reactive sites for the adsorption and conversion of LiPSs, and enhanced the electron/ion transfer rate to ensure efficient ionic/electronic transport. The electrochemical results indicated that the VN@NC-modified separator had a significant part in expediting oxidation–reduction kinetics and increasing cathode sulfur availability. The advantageous electrochemical performance of LSBs constructed with the VN@NC-modified separator has an incipient capacity of 1052.4 mAh g^−1^, and the degradation rate averaged 0.085% over 400 rounds at 1 C. At a low electrolyte/sulfur (E/S) ratio (5 μL mg^−1^) and high sulfur loading (5.41 mg cm^−2^), the LSBs still showed an incipient capacity of 503.5 mAh g^−1^ and were preserved at 461.5 mAh g^−1^ over 100 rounds at 1 C. These further demonstrate the potential application of VN@NC as a separator-modified material to realize high-performance LSBs.

## 2. Experimental Section

### 2.1. Materials

V_2_AlC was obtained from 11 Technology Co., Ltd. (Jilin, China). PVDF (HSV900) was obtained from Arkema Co., Ltd. (Paris, France). Ketjen black (EC-600JD) was obtained from Lion Specialty Chemicals Co., Ltd. (Tokyo, Japan). NaOH (AR), melamine (C_3_H_6_N_6_) (AR), S powder (AR), and *N*-Methylpyrrolidone (NMP) (AR) were obtained from Sinopharm Chemical Reagent Co., Ltd. (Shanghai, China).

### 2.2. Preparation of VN@NC

Typically, fluoropolymer (1 g) was homogeneously mixed with C_3_H_6_N_6_ (0.5 g) and placed at the front end of the corundum boat, and 0.2 g of V_2_AlC MAX was homogeneously placed at the back end of the same corundum boat. It is then enclosed in a corundum tube protected by high-purity argon gas and maintained at 700 °C for 0.5 h. AlF_3_/VN@NC was collected from the back end of the corundum boat after cooling naturally to 25 °C. Subsequently, an alkali-washing procedure was used to remove the AlF_3_, and then the residual alkali on the surface of the material was washed away and dried to obtain VN@NC.

### 2.3. Preparation of the Modified Separator 

At first, a slurry with good consistency was prepared by grinding and mixing VN@NC or AlF_3_/VN@NC with PVDF in a ratio of 9:1 by weight, using NMP as a solvent. Subsequently, the resulting slurry was evenly applied to the separated polypropylene. It was dried in an oven followed by slicing into 19 mm diameter discs to obtain VN@NC/PP or AlF_3_/VN@NC/PP. 

### 2.4. Material Characterization

The physical phases were analyzed by X-ray diffraction (XRD, Cu Ka radiation, Rigaku Ultima IV, Rigaku, Tokyo, Japan) from 2θ = 5–80°, scanning at 15° per minute. The morphological and microstructural aspects were analyzed by scanning electron microscopy (SEM, Phenom Premium, Thermo Fisher Scientific, Waltham, MA, USA), field emission scanning electron microscopy (FESEM, JEOL-6300F, 15 kV, JEOL, Tokyo, Japan), and transmission electron microscopy (TEM, JEOL JEM 2100 system, JEOL, Tokyo, Japan). An X-ray photoelectron spectrometer (XPS, Thermo, Escalab 250xi spectrometer, Thermo Fisher Scientific, Waltham, MA, USA) was used to analyze the valence and chemical states of the element. Brunner–Emmett–Taylor (BET) surface area analysis was performed by nitrogen adsorption/desorption (NOVA 2000, Quantachrome, Boynton Beach, FL, USA). Li_2_S_6_ adsorption was tested by UV-visible absorption spectrophotometry using a UV-2600 analyzer (Shimadzu, Tokyo, Japan). The sulfur concentration in the material was analyzed by thermogravimetric analysis (TGA, DTG-60 h) in a nitrogen atmosphere over a range of 10–800 °C at 10 °C min^−1^.

### 2.5. Electrochemical Characterization

Firstly, sublimated sulfur and Ketjen black (KB) were uniformly milled in a 3:1 ratio of weight for 30 min, followed by transfer into the Teflon reactor and kept at 155 °C for 12 h to form a KB/S composite as a sulfur-hosting material. The cathode slurries were prepared by mixing KB/S, AB, and PVDF uniformly in the ratio of 8:1:1 by weight with NMP as the dispersant, uniformly applied to aluminum foil, which is dried and sliced into 12 mm diameter sheets and applied as a sulfur cathode that contains about 1 mg cm^−2^ of sulfur. The prepared VN@NC/PP, AlF_3_/VN@NC/PP, or PP were employed as the diaphragms and the lithium metal chips were used as the anodes, and the electrolyte was formulated as 1M LiTFSI in DME: DOL = 1:1 Vol% with 1% LiNO_3_. The electrochemical performance of the samples was appraised by assembling button batteries (CR2032) in a glove box. Constant current charge/discharge measurements were performed with the batteries measurement device (Land CT2001A, Wuhan LANDEC Electronic Co., Wuhan, China) with a voltage range from 1.7 to 2.8 V. Cyclic voltammetry (CV) and electrochemical impedance spectroscopy (EIS) were measured with an electrochemical workstation (IviumStat, Ivium technologies BV, Eindhoven, The Netherlands). CV tests were measured at 0.1 mV s^−1^. All electrochemical measurements were carried out at 25 °C.

Assembly of Li_2_S_6_ symmetrical battery and Li_2_S nucleation and dissolution tests: The slurry for the working electrode of the symmetrical battery consisted of VN@NC or AlF_3_/VN@NC with PVDF in a ratio of 9:1 by weight, using NMP as a solvent. These pole pieces were applied as cathode and anode, respectively. The symmetric batteries were constructed from 0.2 M Li_2_S_6_ electrolyte and PP diaphragm. Symmetrical batteries were measured by CV with a voltage interval of −1 to 1 V at 5 mV s^−1^. The nucleation growth and dissolution of Li_2_S were assessed by a CR2032 battery with the same cathode as that of the Li_2_S_6_ symmetric cell and lithium metal flakes at the anode, with 25 μL of 0.2 M Li_2_S_8_ electrolyte added dropwise on the cathode side, and 25 μL of lithium–sulfur electrolyte (1 M LiTFSI in DME: DOL = 1:1 Vol% with 1% LiNO_3_) added dropwise on the anode side.

## 3. Result and Discussion

**Figure 1:**
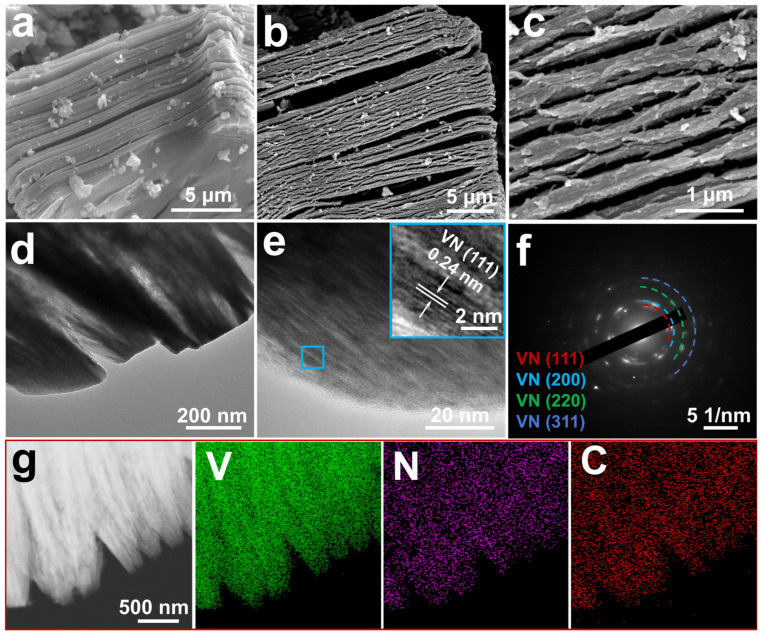
(**a**) FESEM image of AlF_3_/VN@NC; (**b**,**c**) FESEM images of VN@NC; (**d**,**e**) TEM and HRTEM images of VN@NC; (**f**) SAED pattern of VN@NC; (**g**) the corresponding elemental (V, N, C) mapping images of VN@NC.

In the FESEM image (Figure 1a), AlF_3_/VN@NC indicates a significant volume expansion compared to the compact bulk structure of V_2_AlC itself, and a pronounced lamellar structure is produced. Meanwhile, the presence of bulk AlF_3_ particles on and around the AlF_3_/VN@NC layer is observed. The precipitation of AlF_3_ from the interlayer further enlarges the interlayer spacing. As shown in Figure 1b,c, the AlF_3_ has been removed from the surface of the VN@NC material after treatment with alkali solution. In this post-treatment process, the lamellar structure of the VN@NC material became more obvious and still well maintained the carbon skeleton derived from MAX with the carbon layer thickness ranging from 0.15–0.25 μm. The carbon skeleton increases the ionic conductivity of VN@NC, and the greater specific surface area provides abundant catalytically active spots, benefiting the sorption and catalytic transformation of LiPSs. The TEM image (Figure 1d) shows a clear layered structure of VN@NC, with the thickness of the layers corresponding to Figure 1c. Meanwhile, the HRTEM image (Figure 1e) exhibits the growth of VN mainly along the V layer in V_2_AlC, this may be due to the template effect of the MAX, with an inter-lattice stripe distance of 0.24 nm in the inset, which corresponds to the crystalline surface of VN (111). Diffraction rings in selected area electron diffraction (SAED) images (Figure 1f) are associated with the (111), (200), (220), and (311) crystalline facets of the VN, which indicates that VN@NC has a polycrystalline structure. In addition, the element mapping for VN@NC (Figure 1g) demonstrates that V, N, and C are uniformly dispersed in the VN@NC material, which demonstrates that the VN nanoparticles are distributed uniformly in the NC skeleton derived from MAX.

**Figure 2:**
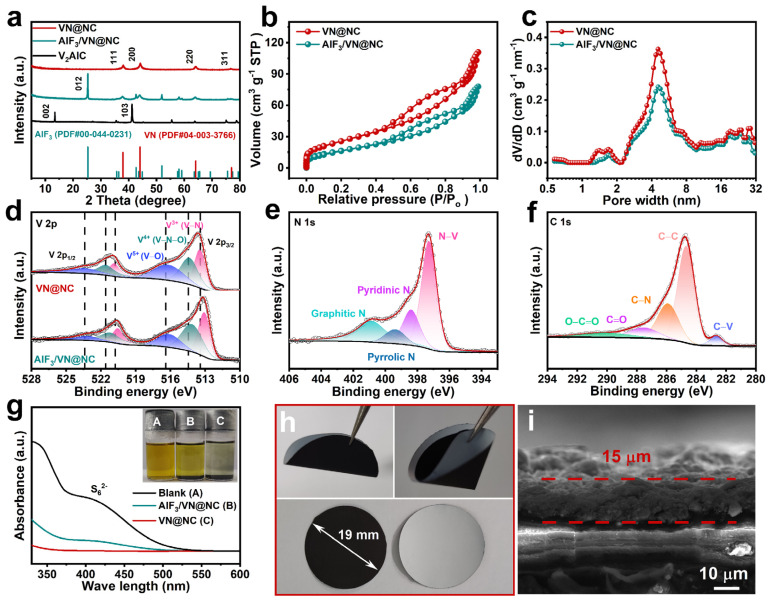
(**a**) XRD patterns for VN@NC, AlF_3_/VN@NC, and V_2_AlC; (**b**,**c**) N_2_ sorption/desorption isotherms and pore size distribution for VN@NC and AlF_3_/VN@NC. High-resolution (**d**) V 2p XPS spectra of VN@NC and AlF_3_/VN@NC; (**e**) N 1s, and (**f**) C 1s XPS spectra of VN@NC; (**g**) digital photo and UV–vis absorption spectra of the Li_2_S_6_ solutions before and after adding AlF_3_/VN@NC and VN@NC; (**h**) digital photos of the VN@NC/PP; (**i**) SEM image of the cross-section of the as-prepared VN@NC/PP.

Typical XRD patterns of VN@NC, AlF_3_/VN@NC, and V_2_AlC are presented in Figure 2a. Compared with the diffraction pattern of the original V_2_AlC MAX, in the diffraction patterns of the AlF_3_/VN@NC samples obtained after high-temperature etching and nitriding treatments, the characteristic peaks (002) and (103) corresponding to V_2_AlC have completely disappeared, and obvious crystalline variations have been observed. A high-intensity characteristic peak corresponding to the AlF_3_ (012) crystal plane (PDF#00-044-0231) appeared at 37.9°, suggesting that fluorine-containing gases produced by pyrolysis of fluoropolymer can selectively react with the Al layer. Simultaneously, VN is generated from the nitridation of the V layer in V_2_AlC by the ammonia produced from melamine pyrolysis. Characteristic peaks attributed to the VN (111), (200), (220), and (311) crystallites (PDF#04-003-3766) can be observed at 37.9°, 44.1°, 64.1°, and 77.0°. While, in the XRD pattern of VN@NC, there are only diffraction peaks attributed to VN (111), (200), (220), and (311) crystal planes, confirming the complete removal of AlF_3_ after treatment with NaOH solution. As shown in Figure 2b, the hysteresis loops are observed in the middle section of the N_2_ adsorption and desorption isotherms of VN@NC and AlF_3_/VN@NC, and the adsorption increases rapidly at lower relative pressures with an upwardly convex curve, which are classic IV-shaped curves. In Figure 2c, the pore sizes of VN@NC and AlF_3_/VN@NC are predominantly mesoporous, and thus both exhibit an obvious adsorption–desorption hysteresis phenomenon in Figure 2b. The specific surface area of AlF3/VN@NC is 42.1 m^2^ g^−1^, which is smaller than that of VN@NC (67.8 m^2^ g^−1^) due to the existence of dense AlF_3_ particles attached on the surface and edge of AlF_3_/VN@NC. In addition, although samples had similar pore sizes before and after alkali washing, the pore volume of VN@NC is 0.154 cm^3^ g^−1^ was higher than that of AlF_3_/VN@NC (0.109 cm^3^ g^−1^), which is favorable for LiPSs adsorption. Even more important, different from VN, the poor conductivity of AlF_3_ is not favorable for the catalytic conversion of LiPSs. After washing off the AlF_3_, the high electrical conductivity and catalytic activity of VN@NC itself synergized with the strong adsorption of polysulfides, accelerated the conversion of LiPSs, and further accelerated the Li_2_S nucleation [28,36]. The valence and chemical states of the elements in the materials are analyzed by XPS. Figure S1 presents the XPS full spectra of VN@NC and AlF_3_/VN@NC, and it is found that the F and Al elements in VN@NC almost completely disappear after heat treatment with alkali solution, which indicates that NaOH has sufficiently reacted with AlF_3_. In an attempt to explore the influence of the thermal processing of alkaline solutions on the V element in VN@NC, we have analyzed the well-resolved V 2p spectra of the two materials. As shown in Figure 2d, in the V 2p spectra, there are two overt characteristic peaks (V 2p_3/2_ and V 2p_1/2_) in the 510 to 528 eV range for VN@NC and AlF_3_/VN@NC. Among them, the characteristic peaks of VN@NC are fitted as three pairs of double peaks located at 513.44/520.85 eV, 514.36/521.58 eV, and 516.17/523.38 eV, which represent the V–N (V^3+^), V–N–O (V^4+^), and V–O (V^5+^) chemical compositions and valence states, respectively. Similarly, the V 2p spectra of AlF_3_/VN@NC is fitted to three pairs of double peaks located at 512.89/520.48 eV, 513.81/521.07 eV, and 515.41/522.5 eV, respectively [56,57]. It can be found that there is a shift of the V 2p of VN@NC towards higher binding energies after heat treatment with alkali solution, and the relative content V^5+^ (V–O) increased, which may be due to the interaction of OH^−^ with vanadium atoms in the solution. As presented in Figure 2e, in the well-resolved N 1s spectra of VN@NC, the features peaks at 397.28, 398.39, 399.38, and 400.99 eV belong to N–V, pyridine N, pyrrole N, and graphite N, respectively, where the intensity of N–V is higher, which suggests that the VN has a better crystallinity [58,59]. In particular, five types of carbon are present in the well-resolved C 1s spectra of VN@NC (Figure 2f): C–V (282.67 eV), C–C (284.69 eV), C–N (285.91 eV), C=O (287.53 eV), and O–C=O (290.1 eV), with the highest relative content being C–C, which contributes to the electrical conductivity of the VN@NC material [58,60,61].

To further explain the effect of morphology and structural design on electrochemical performance, the adsorption capabilities of VN@NC and AlF_3_/VN@NC for Li_2_S_6_ are characterized. As displayed in the digital photo in Figure 2g, the color of the Li_2_S_6_ solution with the addition of AlF_3_/VN@NC material slightly lightened after 9 h of standing, indicating that AlF_3_/VN@NC has some adsorption effect on Li_2_S_6_, but it is not strong enough, which may be caused by the presence of unreactive AlF_3_ on the surface of the material. The Li_2_S_6_ solution with the VN@NC material is significantly lighter in color, which indicates that VN@NC has a strong physicochemical adsorption effect on Li_2_S_6_. The supernatant after adsorption of the materials was analyzed using UV absorption spectroscopy. The broad peak in the 350–450 nm region belongs to the S_6_^2−^, and the peak intensity of the Li_2_S_6_ solution with the addition of AlF_3_/VN@NC is drastically reduced compared with that of the blank Li_2_S_6_ solution, whereas the solution with VN@NC exhibits an even lower absorbed peak intensity, which suggests that VN@NC possesses stronger physicochemical adsorption towards LiPSs, thus effectively suppressing the shuttle effect [14,47]. The PP separator is coated with VN@NC or AlF_3_/VN@NC on one side and then sliced into 19 mm diameter disks with a face load of about 0.9 mg cm^−2^, as shown in Figure 2h. The modified separator still retains excellent flexibility and mechanical properties. The SEM image (Figure 2i) confirmed that a barrier layer (VN@NC and PVDF) of about 15 μm is constructed to suppress the shuttle effect. In addition, the wettability of the modified separators toward the electrolyte is illustrated in Figure S2. A small amount of electrolyte (8 μL) is needed to ensure the sufficient wetting of VN@/PP, which is much lower than AlF_3_/VN@/PP (12 μL) and plain PP (20 μL), indicating the better wettability of modified separator.

**Figure 3:**
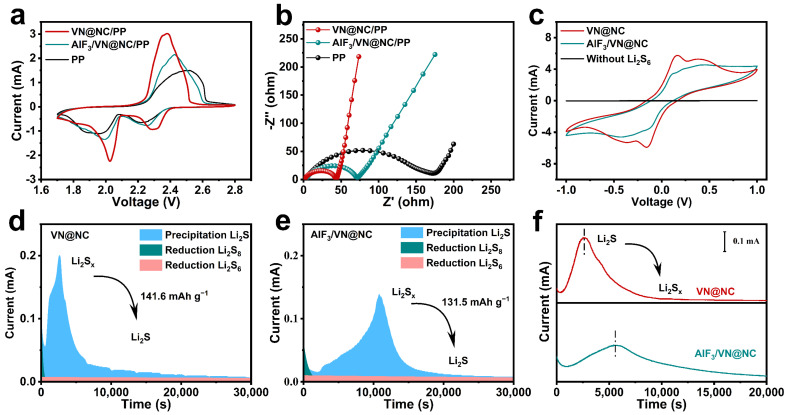
(**a**) CV curves at 0.1 mV s^−1^ and (**b**) EIS spectra of LSBs assembled with different separators; (**c**) CV curves of symmetrical batteries with different electrodes at 5 mV s^−1^; (**d**,**e**) Li_2_S nucleation test of different electrodes; (**f**) Li_2_S dissolution test of different electrodes.

For the investigation of the influences of modified separator on the electrochemical properties of LSBs, we assembled LSBs with KB/S (73.84 wt%, Figure S3) as the cathode and VN@NC/PP, AlF_3_/VN@NC/PP, or PP as the separator. As depicted in Figure 3a, the LSBs were analyzed by CV curves at 0.1 mV s^−1^ within a potential window of 1.7–2.8 V. For VN@NC/PP, two significant reductive peaks at 2.29 V and 2.03 V represent the conversion of sulfur (S_8_) to soluble long-chain LiPSs and the deposition of long-chain LiPSs to form Li_2_S_2_/Li_2_S, respectively, in the discharge stage [62]. The oxidation peak at 2.38 V is associated with the oxidation of Li_2_S_2_/Li_2_S to Li_2_S_4_/Li_2_S_6_ and ultimately to S_8_ [63]. The reductive peaks potential of VN@NC/PP is higher compared to the AlF_3_/VN@NC/PP (2.26 V/2.0 V) and PP diaphragm (2.2 V/1.96 V), and the oxidative peak potential is lower compared to the AlF_3_/VN@NC/PP (2.43 V) and PP (2.51 V), and the peak currents of redox peaks are higher, indicating a faster rate of redox reaction [64]. The above results manifest that VN@NC/PP is effectively accelerating the interconversion of LiPSs with Li_2_S_2_/Li_2_S and promoting the electrochemical reaction kinetics. The electrochemical impedance spectra (EIS) of the different separators are shown in Figure 3b. The EIS curves are composed of semicircles in the high-frequency range and trailing lines in the low-frequency range, which correspond to the charge transfer resistance (R_ct_) and Warburg impedance, respectively [54,65]. The R_ct_ (43.3 Ω) of the LSBs assembled with VN@NC/PP is smaller compared to the Rct (71.0 Ω) with AlF_3_/VN@NC/PP and the R_ct_ (173 Ω) with PP, resulting in a faster charge transmission rate. Meanwhile, the LSBs with VN@NC/PP have a higher gradient in the low-frequency area, which demonstrates the lower ion diffusion resistance of VN@NC/PP during charge/discharge cycling, which facilitates lithium-ion diffusion and transport, resulting in outstanding rate performance. The electrocatalytic activities of VN@NC and AlF_3_/VN@NC are evaluated by symmetric batteries. The symmetric batteries are assembled with Li_2_S_6_ as the electrolyte, and the CV tests are performed in the voltage range of −1–1 V at 5 mV s^−1^. Two pairs of redox peaks are present on the CV curve of VN@NC in Figure 3c, which are symmetrically situated around −0.16/0.16 V and −0.38/0.38 V, respectively, demonstrating a progressive LiPSs conversion reaction and a higher peak current response compared with that of AlF_3_/VN@NC, which suggests an advanced LiPSs conversion capability [13,66].

Chronoamperometry technology is applied to validate the catalytic properties of VN@NG and AlF_3_/VN@NG and on the Li_2_S nucleation and dissolution process. The constant potential time–current curves of Li_2_S deposited on VN@NC and AlF_3_/VN@NC surfaces are shown in Figure 3d,e, respectively. The Li_2_S precipitation capacity of VN@NC is 141.6 mAh g^−1^, which is higher compared to the 131.5 mAh g^−1^ of AlF_3_/VN@NC, and VN@NC can provide a higher constant potential current and faster Li_2_S nucleation response time. It is indicated that VN@NC remarkably enhances the process of nucleation and growth of Li_2_S, which assists to hasten the rapid transformation of LiPSs to Li_2_S and suppress the shuttling effect [67,68]. An Li_2_S dissolution test is further conducted to investigate the superiority of VN@NC in improving the reverse reactions. As shown in Figure 3f, VN@NC exhibits a higher constant potential current with a faster response time for Li_2_S dissolution compared to AlF_3_/VN@NC, which implies that VN@NC can effectively reduce the overpotential for Li_2_S oxidation and thus facilitate the Li_2_S-to-LiPSs conversion process [69,70]. It can be observed that VN@NC exhibits outstanding catalytic properties for both Li_2_S precipitation and dissolution, which will strongly facilitate the acceleration of the reaction kinetics during the interconversion process between LiPSs and Li_2_S. Moreover, the shuttle effect is strongly suppressed, thus realizing the enhancement to the whole of LSBs.

**Figure 4:**
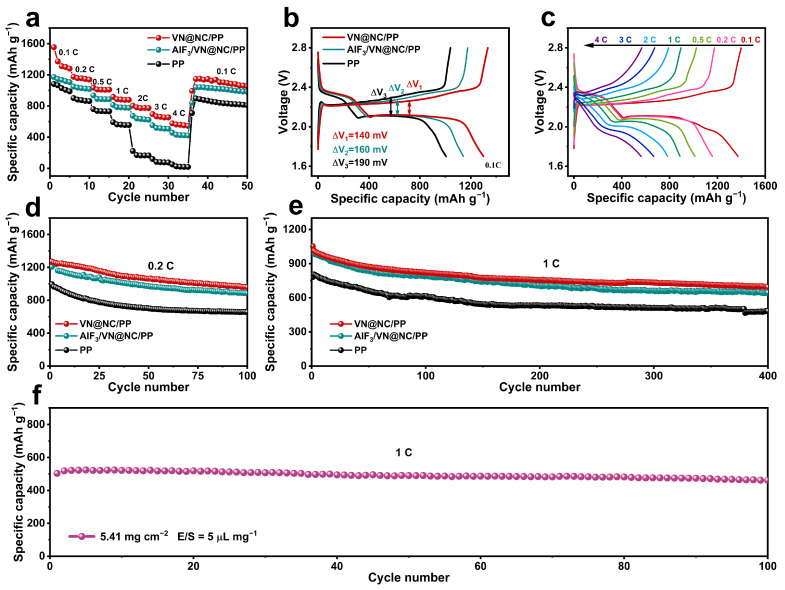
(**a**) Rate performances of LSBs with different separators; (**b**) the charge–discharge profiles of batteries with different separators at 0.1 C. (**c**) the charge–discharge of battery with VN@NC/PP at varied current densities; (**d**) cycling performances at 0.2 C for 100 cycles; (**e**) long cyclic performances at 1 C for 400 cycles; (**f**) long cyclic performance of VN@NC/PP under a low E/S ratio of 5 μL mg^−1^ and a high surface loading of sulfur of 5.41 mg cm^−2^ at 1 C.

The rate and cycling performances of LSBs assembled with VN@NC/PP, AlF_3_/VN@NC/PP, or PP are tested under different current densities. As presented in Figure 4a, the capacities of VN@NC/PP are 1372, 1155, 1010, 823, 783, 660, and 560 mAh g^−1^ at 0.1, 0.2, 0.5, 1, 2, 3, and 4 C, respectively. In contrast, the rate capacities of AlF_3_/VN@NC/PP or PP are lower. The higher structural and chemical stability and better sulfur utilization of VN@NC/PP are illustrated [71]. The constant current discharge–charge curves of VN@NC/PP, AlF_3_/VN@NC/PP, and PP at 0.1 C for the third cycle are selected (Figure 4b). The corresponding overpotential in the charge–discharge curve is noted as ΔE. ΔE_1_ = 140 mV for VN@NC/PP is lower compared to AlF_3_/VN@NC/PP (ΔE_2_ = 160 mV) and PP (ΔE_3_ = 190 mV). As shown in Figure 4c, for LSBs with VN@NC/PP, the discharge–charge curves are selected for the third cycle under 0.1 to 4 C. As the current density increases, the voltage polarization phenomenon is more obvious, but the discharge platform remains comparatively stable, and a platform for the transformation from long-chain LiPSs to Li_2_S_2_/Li_2_S can still be seen at high current densities of 4 C. The above results demonstrate that the special physicochemical properties of VN@NC play an important role in improving the oxidation–reduction kinetics, reducing polarization, and improving the rate performance of the cathode [31,62]. Figure 4d illustrates the cycle performances of VN@NC/PP, AlF_3_/VN@NC/PP, and PP at 0.2 C. The incipient capacity of VN@NC/PP is 1271 mAh g^−1^ and remained at 960 mAh g^−1^ over 100 rounds. The averaged capacity degradation ratio of 0.24% per round is lower compared to AlF_3_/VN@NC/PP (0.27%) and PP (0.34%). Meanwhile, at 0.5 C, the incipient discharge capacity of the LSBs with VN@NC/PP is 1189.8 mAh g^−1^ and remains at 754 mAh g^−1^ over 300 rounds, while the incipient capacities of AlF_3_/VN@NC/PP and PP are 1089 mAh g^−1^ and 870.4 mAh g^−1^, respectively, and decayed to 697.8 mAh g^−1^ and 582.6 mAh g^−1^ over 300 rounds, respectively (Figure S4). As shown in Figure 4e, at 1 C, the incipient capacity of VN@NC/PP is 1052.4 mAh g^−1^ and remains 693.8 mAh g^−1^ over 400 rounds with an average degradation rate of 0.085% per rounds, shows superior cycling stability to AlF_3_/VN@NC/PP (646 mAh g^−1^, 0.094%) and PP (483 mAh g^−1^, 0.095%).

To verify the effectiveness of the modified separator in anchoring polysulfides and suppressing the shuttle effect in practical applications, the batteries after cycling were disassembled and analyzed. As shown in Figure S5, yellow LiPSs are apparent on the PP separator and visible on the corresponding lithium anode surface, suggesting that LiPSs have shuttled across the separator. The LiPSs are also observed on the AlF_3_/VN@NC/PP; however, they are hardly observed on the surface of VN@NC/PP. The above findings demonstrate that VN@NC/PP has a stronger barrier effect on LiPSs, suppressing the shuttling effect significantly. To envisage practical applications, we test the electrochemical performance of LSBs based on VN@NC/PP at a low E/S ratio of 5 μL mg^−1^ and a high surface loading of sulfur of 5.41 mg cm^−2^ (Figure 4f). The LSBs still represent an incipient capacity of 503.5 mAh g^−1^ and maintain at 461.5 mAh g^−1^ over 100 rounds at 1C. It is important to note that LSBs at low E/S ratios and high sulfur-loading conditions have lower capacity and lower degradation rates, which is caused by less electrolytes, allowing gradual sulfur utilization, lower overall sulfur availability, and fewer LiPSs dissolved in the electrolyte [72,73]. In comparison with other LSBs with modified separators, the electrochemical performance of cells with VN@NC/PP is competitive, as shown in Table S1 [74,75,76,77,78,79,80,81].

## 4. Conclusions

In summary, we prepare VN@NC material by the strategy of etching and high-temperature nitriding of V_2_AlC MAX and apply it as a separator-modified material in LSBs. Thanks to the outstanding catalytic performance of VN and the superior electrical conductivity of a carbon skeleton derived from MAX, the synergistic effect between the two significantly suppresses the shuttle effect and increases cathode sulfur availability. The LSBs with VN@NC/PP exhibit an incipient capacity of 1052.4 mAh g^−1^ at 1 C and remain at 693.8 mAh g^−1^ over 400 rounds, with an average degradation rate for every cycle of only 0.085%, reflecting outstanding cycle stability. The assembled LSBs also demonstrate good cycling stability with a low E/S ratio (5 μL mg^−1^) and high surface-loaded sulfur (5.41 mg cm^−2^) as an incipient capacity of 503.5 mAh g^−1^ is achieved and remains 461.5 mAh g^−1^ over 100 rounds at 1 C. The strategy of VN nanoparticles grown in situ on an N-doped carbon skeleton presents potential applications in high-performance LSBs. A remarkably new way of preparing MAX-derived-modified materials for separators is provided to realize high-performance LSBs.

## Data Availability

Data is contained within the article or supplementary material.

## Supplementary Materials

The following supporting information can be downloaded at: https://www.mdpi.com/article/10.3390/nano14080656/s1, Figure S1. XPS survey spectrum of VN@NC and AlF_3_/VN@NC; Figure S2. Digital photographs of the amount of electrolyte required to wet the VN@NC/PP, AlF_3_/VN@NC/PP, and PP separators; Figure S3. TG diagram of KB/S for checking the sulfur loading; Figure S4. (a) Charge-discharge curves of the first cycle and (b) cycling stability for the Li-S batteries with different separators at 0.5 C; Figure S5. Digital images of the cathode, separators, and anode of lithium-sulfur batteries with VN@NC/PP, AlF_3_/VN@NC/PP, and PP separator after deep cycling; Table S1: Comparison of key performance parameters of different diaphragm modification materials for lithium-sulfur batteries [74,75,76,77,78,79,80,81].
