# Supplementary material for: Multifunctional Vanadium Nitride-Modified Separator for High-Performance Lithium–Sulfur Batteries
Source: Nanomaterials (Basel). 2024 Apr 10;14(8):656. doi: 10.3390/nano14080656 (PMC11053798; doi:10.3390/nano14080656)
Supplement: Supplementary file 1 [file nanomaterials-14-00656-s001.zip › nanomaterials-2935811-supplementary.pdf]

Supporting Information

Article

Multifunctional Vanadium Nitride-Modified Separator for High-Performance Lithium–Sulfur Batteries

Sen Liu, Yang Liu *, Xu Zhang, Maoqiang Shen, Xuesen Liu, Xinyue Gao, Linrui Hou and Changzhou Yuan *

School of Materials Science & Engineering, University of Jinan, Jinan 250022, China; liusen@stu.ujn.edu.cn (S.L.); zhangxu@stu.ujn.edu.cn (X.Z.); shenmq@stu.ujn.edu.cn (M.S.); liuxs@stu.ujn.edu.cn (X.L.); gaoxinyue@stu.ujn.edu.cn (X.G.); mse_houlr@ujn.edu.cn (L.H.)

* Correspondence: mse_liuy@ujn.edu.cn (Y.L.); ayuancz@163.com or mse_yuancz@ujn.edu.cn (C.Y.)

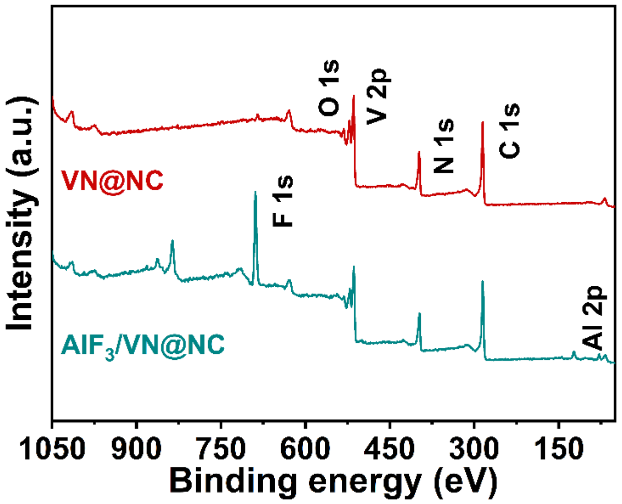

Figure S1. XPS survey spectrum of VN@NC and AlF_3/VN@NC.

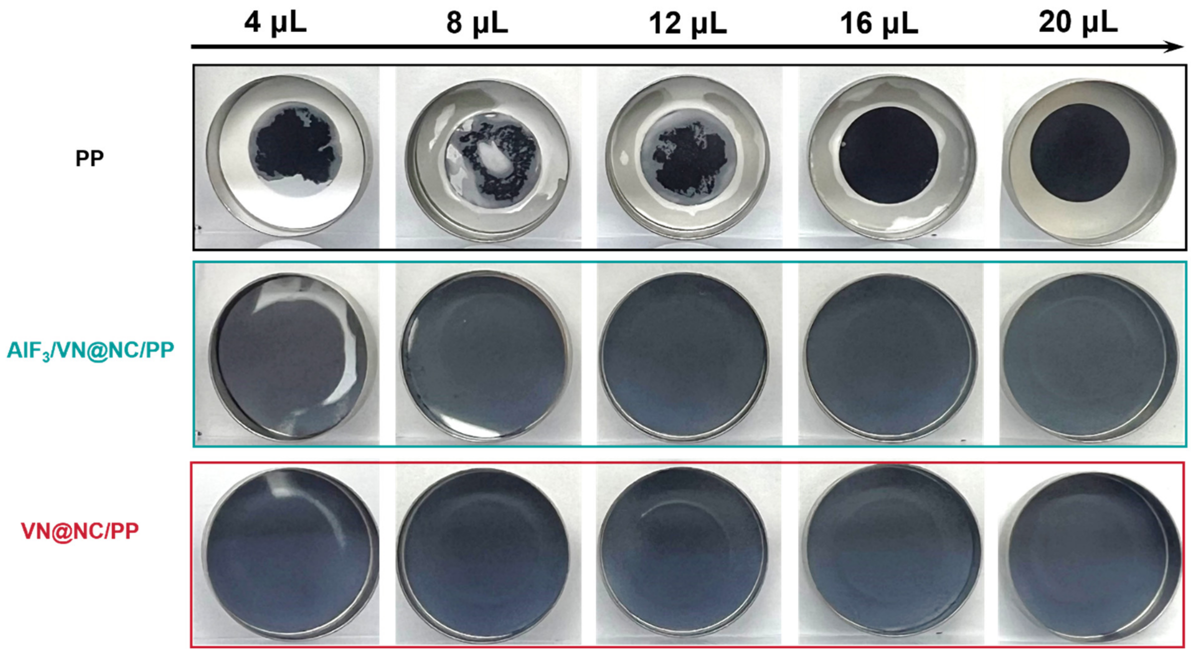

Figure S2. Digital photographs of the amount of electrolyte required to wet the VN@NC/PP, AlF_3/VN@NC/PP, and PP separators

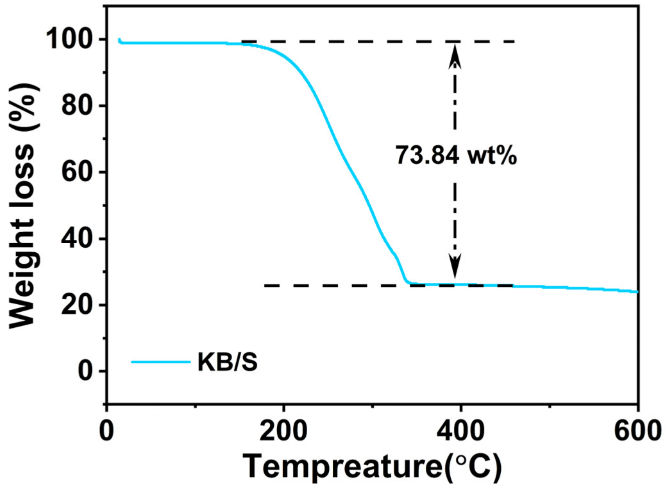

Figure S3. TG diagram of KB/S for checking the sulfur loading.

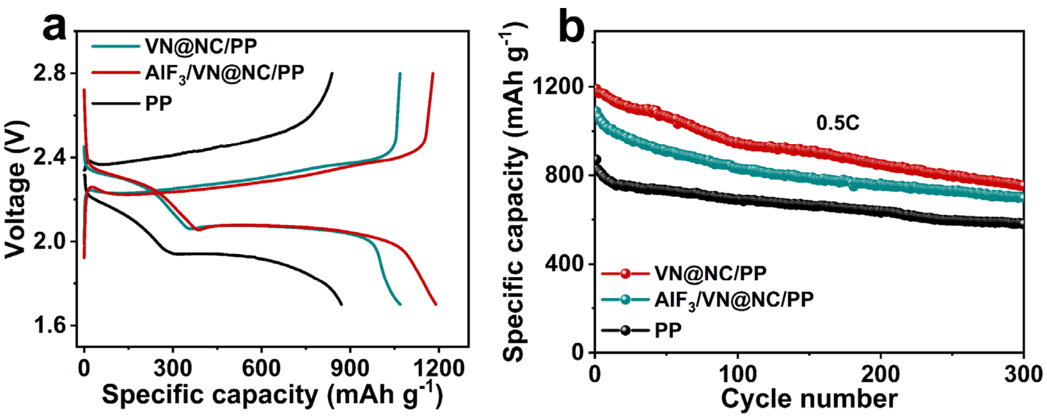

Figure S4. **(a)** Charge-discharge curves of the first cycle and **(b)** cycling stability for the Li-S batteries with different separators at 0.5 C.

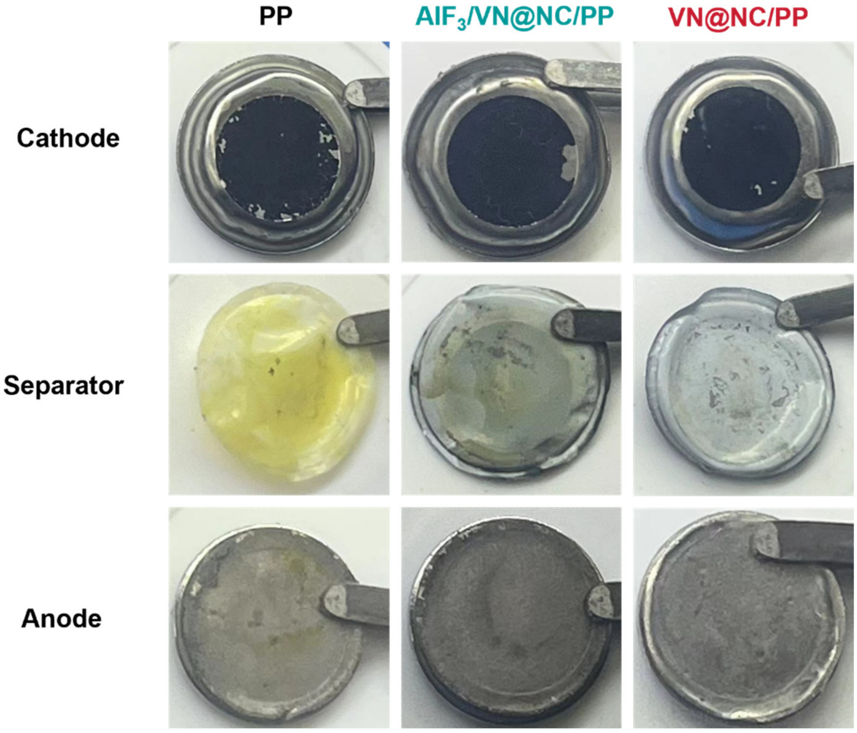

Figure S5. Digital images of the cathode, separators, and anode of lithium-sulfur batteries with VN@NC/PP, AlF_3/VN@NC/PP, and PP separator after deep cycling.

Table S1. Comparison of key performance parameters of different diaphragm modification materials for lithium-sulfur batteries.

separator	sulfur-loading (mg cm^{-2})	Initial capacity (mA h g^{-1})	Life (Cycling number)	current density (C)	decay/cycle	ref.
VN@NC	~1.0<br>~5.41	1052.4<br>503.5	400<br>100	1<br>1	0.085%<br>0.083%	this work<br>this work
NSPCF@CoS_2@C-150	-<br>3.0	846.1<br>668.5	100<br>100	0.5<br>0.5	0.21%<br>0.25%	[74]
Ta_4C_3-Ta_2O_5	~0.9	801.9	500	1	0.086%	[75]
ZIF-67/SA-PAN	1<br>5.45	801.8<br>-	500<br>100	1<br>0.1	0.089%<br>0.098%	[76]
TpPa-SO_3H/Celgard	1<br>5.4	887<br>788	100<br>100	0.2<br>0.2	0.42%<br>0.16%	[77]
Co_3O_4@GC/N-CNT NF-coated separator	2.0	1187	250	0.1	0.16%	[78]
Al-CPP/Celgard	~1	935.7	500	1	0.12%	[79]
N-C@TiO_2/HNC-coated separator	1.64	1373	500	0.1	0.11%	[80]
CNT CoN-V_N	2.5	851.6	250	2	0.09 %	[81]
